# Supplementary material for: Complexome profiling of the Chlamydomonas psb28 mutant reveals TEF5 as an early PSII assembly factor
Source: Plant Cell. 2025 Mar 17;37(6):koaf055. doi: 10.1093/plcell/koaf055 (PMC12164590; doi:10.1093/plcell/koaf055)
Supplement: koaf055_Supplementary_Data [file koaf055_supplementary_data.zip › psb28_interactive.zip!/plots/intensity/qualityPlots/peptides/LCI24_Cre03.g179800.t1.2.html]

### Cre03.g179800.t1.2

Trivial name: LCI24  
SeqCoverage [median]: 5.70  
Mass: 18.10 kDa  
  
 

| Sequence | MissedCleavage | Mass [Da] | wtIntensity | muIntensity | Score |
| --- | --- | --- | --- | --- | --- |
| VGGSSGFSAR | 0 | 923.45 | 57404.92 | 90078.55 | 172.94 |
|  |  |  | 57404.9 | 90078.6 |  |
